# Are We Consulting, Sharing Care, or Taking Over? A Conceptual Framework

**DOI:** 10.1089/pmr.2023.0079

**Published:** 2024-02-23

**Authors:** José Pereira, Christopher Klinger, Hsien Seow, Denise Marshall, Leonie Herx

**Affiliations:** ^1^ Division of Palliative Care, Department of Family Medicine, McMaster University, Hamilton, Ontario, Canada.; ^2^ Faculty of Medicine, University of Navarra, Pamplona, Navarra, Spain.; ^3^ Pallium Canada, Ottawa, Ontario, Canada.; ^4^ Department of Oncology, McMaster University, Hamilton, Ontario, Canada.; ^5^ Department of Pediatrics, University of Calgary, Calgary, Alberta, Canada.

**Keywords:** consultation, palliative care, primary care, service models, shared care, specialist

## Abstract

**Background:**

Primary- and specialist-level palliative care services are needed. They should work collaboratively and synergistically. Although several service models have been described, these remain open to different interpretations and deployment.

**Aim:**

This article describes a conceptual framework, the Consultation-Shared Care-Takeover (C-S-T) Framework, its evolution and its applications.

**Design:**

An iterative process informed the development of the Framework. This included a symposium, literature searches, results from three studies, and real-life applications.

**Results:**

The C-S-T Framework represents a spectrum anchored by the *Consultation* model at one end, the *Takeover* model at the other end, and the *Shared Care* model in the center. Indicators, divided into five domains, help differentiate one model from the other. The domains are (1) Scope (What aspects of care are addressed by the palliative care clinician?); (2) Prescriber (Who prescribes the treatments?); (3) Communication (What communication occurs between the palliative care clinician and the patient's attending clinician?); (4) Follow-up (Who provides the follow-up visits and what is their frequency?); and (5) Most responsible practitioner (MRP) (Who is identified as MRP?). Each model demonstrates strengths, limitations, uses, and roles.

**Conclusions:**

The C-S-T Framework can be used to better describe, understand, assess, and monitor models being used by specialist palliative care teams in their interactions with primary care providers and other specialist services. Large studies are needed to test the application of the Framework on a broader scale in health care systems.

## Background

The World Health Organization (WHO) and other experts have highlighted the need for both primary- and specialist-level palliative care services in every country, working synergistically to meet the needs of persons with serious illnesses who could benefit from palliative care.^1–4^

Specialist palliative care is provided by clinicians and teams with advanced expertise in palliative care, allowing them to care for patients with complex needs, provide palliative care education, undertake research, and provide leadership in the field.^5,6^ Examples of specialist palliative care services include palliative care units and community- and hospital-based support teams. Gaps in these services, including a scarcity of palliative care specialists, have been noted.^7,8^

However, the palliative care needs of a population cannot be addressed solely by palliative care specialists,^6,9^ especially if palliative care needs to be available across cancer and noncancer illnesses, activated earlier in the illness trajectory, and present across all care settings.^10–18^ Hence, the growing call for primary-level palliative care provision—also referred to as generalist-level palliative care—alongside specialist palliative care services.^6,15,19^ If equipped with core palliative care competencies and supported by specialist palliative care teams, health care professionals across many professions and specialty areas could provide a palliative care approach for their patients, thereby increasing palliative care access across all care settings.^20,21^ The components of primary palliative care have been elaborated.^15,22,23^

Gomez-Batiste and colleagues have recommended that the role of specialized palliative care services is to train and support, providing care only for complex cases.^3^ They call for flexible and cooperative partnership models between specialist- and primary-level palliative care providers.^3^ Collaboration constitutes a key component in many strategies and models. Integrated, consultation, liaison, pop-up, shared care, team-based, and trajectory models described by Luckett and colleagues, for example, incorporate collaboration between palliative care specialists and other services.^2^ Various definitions and descriptions of each of these models have been proposed and some overlap.^2,24^

Lack of clarity presents a challenge when describing, planning, promoting, monitoring, and auditing palliative care services. Consultation and shared care models, for example, are subject to nuances and open to different interpretations and operationalization.^25^ Similarly for integrated, liaison, and pop-up models, the model or approach adopted may have significant impact on the delivery of, and access to, palliative care on the short- and long-term.

This article describes the development, evolution, and applications of a conceptual framework, called the Consultation-Shared Care-Takeover (C-S-T) Framework, that facilitates the understanding, description, assessment, and monitoring of how a specialist palliative care clinician or team engages and collaborates with primary care providers and other specialist teams who refer to them.

## Methods

### Overall design and process

The C-S-T Framework has evolved iteratively over several years, with a variety of activities contributing to its genesis and evolution.

### Early development

The main development of the framework started in 2012 with the drafting of an early version. However, this early version was informed by earlier experiences and literature. In 2001 and 2002, Pallium Canada, a Canadian nonprofit organization that advances primary palliative care, organized symposia with palliative care and primary care leaders to explore strategies to build primary palliative care capacity.^26^ Consultation and shared care models were emerging as best practices.^27–29^ Reflections by an in-hospital palliative care service in Switzerland on what the team's role relative to other services should be, provided additional insights.^30–33^

### 2012 symposium in Ottawa, Canada

In 2012, a two-day symposium (led by the authors) was convened in Ottawa, Canada, to explore the face validity and usefulness of an emerging conceptual framework that included three models along a scale, namely *Consultation*, *Shared Care,* and *Takeover*. Canadian and international subject matter experts explored questions such as “Do these models exist in everyday practice?,” “What are their characteristics?,” “What influences their adoption and application?” and “What is their impact?.”^26^

The deliberations identified areas of consensus and disagreement. There was agreement that the three models existed. However, because each included varying degrees of application and nuances, it was felt that they would best be depicted as anchors along a spectrum. For example, while consultation can involve a single or a limited number of visits by the consultant, sometimes multiple follow-up visits and more active prescribing by the consultant may be needed. There was agreement that all three models have their respective roles, strengths, and limitations, depending on the context and goals at hand. There was agreement that a team may at times have to apply different models at different times for different situations. However, it was recognized that excessive flexibility and inconsistency in the application of roles risk creating confusion. There was also agreement that the Framework could help describe, plan, and monitor services. Some indicators that could help differentiate one model from another were proposed and were incorporated in the Framework.

Significant debate occurred around who the “client” is relative to the specialist team, as well as who the most responsible practitioner (MRP) in a shared care model is. While patients and their needs are at the center of care, the referring clinician and attending service also benefit from the consultant's attention. Some participants felt that two clinicians could serve simultaneously as MRPs, each overseeing their specific area of responsibility, but others felt that there could only be one MRP at any given time to avoid confusion and ensure patient safety. There was an agreement that the frequency of follow-up visits should depend on the patient's needs and medical issues, no matter the model.

### Studies

The Framework was applied directly or indirectly in three studies. Brown and colleagues used it in a large population-level study to identify how physicians were providing palliative care to decedents in their last year of life in Ontario, Canada.^34^ Billing codes submitted by clinicians, and a formula previously developed to differentiate between physicians with a palliative care focused practice (specialists) versus those who provided some palliative care as part of their generalist practices (generalists) were used.^35^ Four major patterns were identified: 53% of decedents received no physician-based palliative care; 21.2% received only generalist palliative care; 14.7% received consultation-type palliative care (namely care from both specialists and generalists); and 11.1% received only specialist palliative care (palliative care provided solely by palliative care specialist clinicians).

Maybee and colleagues studied practice models of community-based palliative care clinicians in Ontario.^36^ The goal was primarily to describe their day-to-day work processes, including how they interacted with primary care teams and their motivations for adopting the approaches they used. At the very end of each interview, participants were shown the Framework. The participants endorsed it and felt that it reflected their respective practices. Of the 14 study participants, 4 worked in a *Consultation* model, 8 in a *Takeover* model, and 2 were transitioning to a *Consultation* model. None were found to be using a *Shared Care* model. While all clinicians worked to improve patient care, in the *Takeover* model, participants were primarily motivated by their relationships with patients, and in the *Consultation* model, they were motivated by supporting and building primary-level palliative care.

In a separate study, researchers studying the impact of palliative care physicians with added certificates of competency in palliative care across several community sites in Canada, confirmed practice patterns that aligned with the three models (*Consultation*, *Shared Care,* and *Takeover*).^37^ Funding models and other structures were perceived as incentivizing the *Takeover* model.

### Health services planning and program reviews

The C-S-T Framework has been used to inform strategic planning in some Canadian jurisdictions. It helped inform key elements of the Palliative Care Health Services Delivery Framework in Ontario in 2019^38^ and was also used by Pallium Canada and the Province of New Brunswick's Health Ministry in 2019 to inform the development of primary palliative care capacity.

An external review of palliative care services in a large Canadian region was undertaken by a team that included three of the authors (personal communication). The Framework was used to guide discussions and identify and understand different patterns of practice among community- and hospital-based palliative care teams. Two teams, for example, shifted from *Consultation* to *Takeover* models despite being considered consultation services: one practising exclusively in a *Takeover* model and the other predominantly *Takeover* with some *Consultation*. The transition started over six years previously and coincided with changes in the physician funding model (from salary-type to fee-for-service). To manage increased workloads, one of the teams limited its referral criteria to only patients with significantly reduced functional status and not receiving disease-modifying treatments. Discussions with primary care leaders indicated that few family physicians and primary care teams in the urban parts of the region provided primary palliative care.

### Literature searches

Periodic literature searches of palliative care and other health care-focused publications have contributed to the evolution of the Framework. These publications are referenced throughout this article.

## Results

### The C-S-T Framework: Summary description

**FIG. 1.**
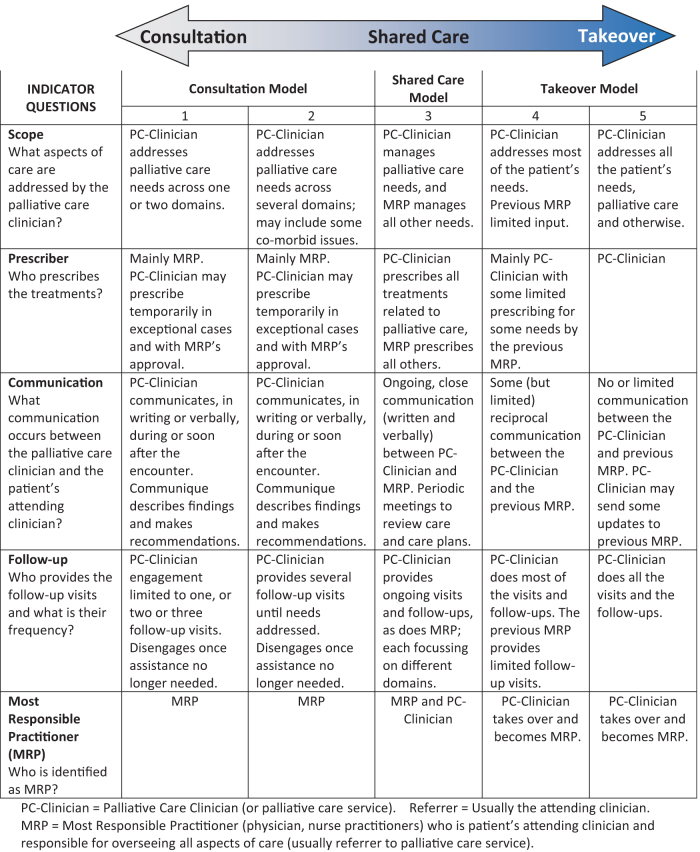
The C-S-T conceptual framework. C-S-T, Consultation—Shared Care—Takeover.

The C-S-T Framework represents a spectrum anchored by the *Consultation* model at one end, the *Takeover* model at the other end, and the *Shared Care* model in the center (Fig. 1).

A table that helps identify which model is applicable is provided. The table lists five key domains that help differentiate one model from another. Each of these is framed as a question. Each question in turn includes descriptors or indicators that further help identify the model that is most applicable. The key questions and their respective domains are (1) Scope (What aspects of care are addressed by the palliative care clinician?); (2) Prescriber (Who prescribes the treatments?); (3) Communication (What communication occurs between the palliative care clinician and the patient's attending clinician?); (4) Follow-up (Who provides the follow-up visits and what is their frequency?); and (5) MRP (Who is identified as MRP?). The initial Framework (2012) listed up to nine potential indicators, but this was reduced through iterative work to five key ones. The earlier version (2012 Symposium) had also included nine subcategories, three each for Consultation, Shared Care, and Consultation. Subsequent work revealed that this created confusion and could be simplified to five subcategories. Moreover, the studies began to reveal that the *Shared Care* model was relatively uncommon and difficult to apply and could be represented adequately by one category.^36,37^

The *Consultation* and *Takeover* models each have two subcategories. This recognizes that within each of these are varying degrees of application and nuances. To facilitate classification, each of the five categories are labeled with a number from 1 to 5; the *Consultation* model is assigned 1 and 2, *Shared Care* 3, and *Takeover* 4 and 5.

### Consultation model description

There are various definitions of *Consultation*. This Framework uses the one by Penrod et al.^39^ and Luckett and colleagues,^2^ which describe consultation as “an approach to care by which specialist advice is provided on assessment and treatment of symptoms, communication about goals of care and support for complex medical decision-making, provision of practical and psychosocial support, care coordination and continuity, and bereavement services when appropriate.” Importantly, they go on to state that “advice is provided without assuming primary responsibility for care, although there is negotiation of the level of palliative care involvement.” This is emphasized by others.^40^

The goal of *Consultation* is to address the needs of the patient, while also supporting the MRP and the attending team.^41^ Advice can be provided without the specialist being directly involved in care.^42^

In some situations, a more limited consultation suffices. The consultant focuses only on the needs identified by the referrer, does a single visit (or one or two follow-up visits), and recommends treatments rather than prescribes. Other situations require a broader consultation in which the consultant addresses multiple domains and follows up with as many visits as are needed. Consultants should sign off, including in writing, when the reasons for referral have been addressed or stabilized, but be available for re-referral should the need arise.

*Consultation* generally requires that the consultant clarify what the attending service is asking for help with. No direct care should be provided unless specifically requested or negotiated.^43^ Von Gunten recommends this “at least until one becomes acquainted with local consult culture and the preferences of individual referring physicians and teams.”^43^ Others place less focus on formally defining a specific question and suggest writing orders when the referring physician is not comfortable doing so or cannot prescribe in a timely manner.^44^

The need for some flexibility is highlighted in *Consultation*.^43^ The expectations of referring practitioners and their preferences regarding the role of the consultant may differ considerably.^43–45^ Moreover, it is not unusual for consultants to identify previously unrecognized or unreported needs that require attention.^46,47^ In general, whether or not consultants write orders depend on the arrangements with the referring practitioners and patients' needs, which should also dictate the frequency and duration of follow-up.

Best practices in consultation have been published.^40,41,43,44,48–53^ Bates, for example, proposed that the ideal consultant will “render a report that informs without patronizing, educates without lecturing, directs without ordering, and solves the problem without making the referring physician feel incompetent.”^41^ The American Medical Association (AMA) has described nine ethical principles pertaining to consultation; three relate to the referring physician and six to the consultant.^54^ Gardiner and colleagues described effective partnerships between specialist palliative care services and generalists.^52^

### Shared care model description

Shared Care was initially described in mental health care and addiction medicine.^28,55,56^ More recently, it is gaining attention in the management of chronic diseases.^57,58^ There is no one single definition of *Shared Care*, and there are various interpretations of it.^59,60^ Not surprisingly, including in palliative care circles, the term is often used loosely. Chomik, quoting Moorehead, defines it as “using the skills and knowledge of a range of health professionals who share joint responsibility in relation to an individual's care.”^56,59^ Overall, there is consensus that *Shared Care* requires that the roles of the various care providers be clearly delineated from the outset and that communication occurs on an ongoing basis. The importance of shared monitoring as well as exchanging patient data through things such as shared health records and data transfers is stressed in this model.^59^ Information exchange should be over and above routine referral and visit notes. *Shared Care* models have been described in palliative care, but closer inspection may reveal them to be more broad consultation models.^29,61^

### Takeover model description

From the perspective of the specialist palliative care clinician or team, *Takeover* occurs when they take over as MRP from the previous clinician, assuming sole responsibility for managing and overseeing all aspects of care. There is no further need for close collaboration and communication, unless the *Takeover* is temporary, and the plan is for the previous attending to reassume the MRP role at some point in the future. The *Takeover* model is most appropriate where the patient's needs are complex and exceed the skills or comfort of the attending clinician.

### Strengths, limitations, and roles of the different models

Each of the three models have their respective strengths, limitations, and roles, depending on the context.^36^ All three contribute to patient care.^2,24,62^ A key strength of the *Consultation* model is its amplifying effect; the expertise of a small group of specialized individualized can be spread to a larger number of patients and health care providers. On the other hand, if *Consultation* is applied too narrowly, and with no flexibility, some patients will not have needs addressed. It requires reciprocity from the MRP who needs to take ownership of providing primary palliative care, which includes after-hours coverage and, in the case of primary care, home visits when needed.

The *Shared Care* model, although meant to harness the respective strengths of each, may be more challenging to operationalize in palliative care where the lines that separate responsibilities may be difficult to draw, especially in end-of-life care.^36,37^ Lack of clarity of roles and suboptimal communication between providers risks optimal patient care. Some regulatory and professional bodies therefore recommend that there be only one MRP at any given time, and that any changes in responsibility be mutually agreed upon and clearly communicated and documented.^63,64^ Despite these challenges, *Shared Care* holds promise for the provision of primary palliative care within multidisciplinary primary care teams.

The *Takeover* model is required when a patient's needs are complex. It may also be required when a patient has no primary practitioner, a growing reality in some jurisdictions.^65^ This model is efficient from the perspective of the palliative care clinician who need not negotiate roles and care plans with another practitioner, and it avoids frustration when recommendations are not implemented by the MRP.^43,52,62,66^ However, primary palliative care is undermined if *Takeover* becomes the predominant model, where palliative care specialists provide both specialist-level and primary-level palliative care. A vicious cycle ensues. Primary care practitioners and other specialists will not acquire or maintain palliative care skills as they increasingly rely on the palliative care service to provide all palliative care. Moreover, the message to them, implied or explicit, is that palliative care can only be done by clinicians with advanced skills in it.^67,68^

### Framework utilization

The Framework is primarily intended as a tool that helps palliative care services reflect, plan, organize, monitor, or audit their interactions with primary care providers and other specialist services. It can be applied at a variety of levels: individual referrals, series of referrals, individual clinicians, or the whole service. At the referral level, every case or a series of consecutive referrals may be analyzed to identify which model was used. Similarly, an individual clinician or a whole team may reflect on what model or models they tend to use and what the dominant one is.

**FIG. 2.**
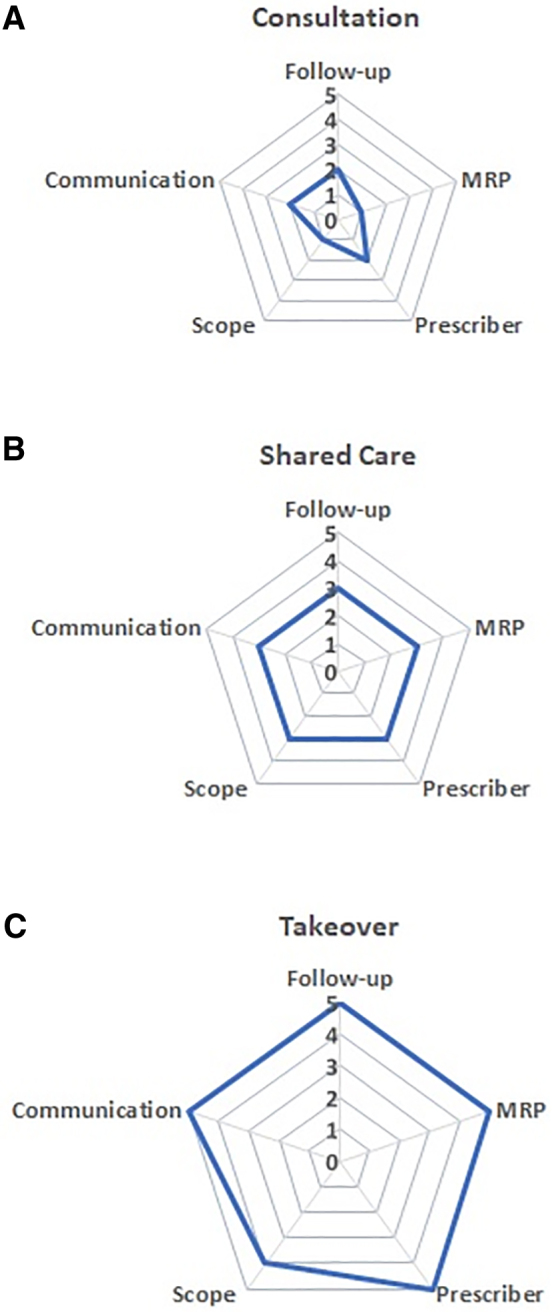
Radar graphs illustrating three different practice models used by three different teams as based on the C-S-T Framework indicators. **(A)** Consultation model. **(B)** Shared care model. **(C)** Takeover model.

A number from 1 to 5 is assigned as per the indicator descriptions for each of the five domains (Scope, Prescriber, Communication, Follow-up, and MRP). The numbers assigned may be homogenous across the five domains. Five “2”s, for example, would denote broader *Consultation*. However, different numbers may sometimes be assigned, such as four 1s and a 2. In these cases, the mode would indicate the most dominant model. Further reflection would be needed if numbers from different sides of the spectrum are assigned simultaneously; one cannot practice both *Consultation* and *Takeover* on the same case at the same time. The results may also be graphed on to a radar chart (see Fig. 2 for an illustration).

**FIG. 3.**
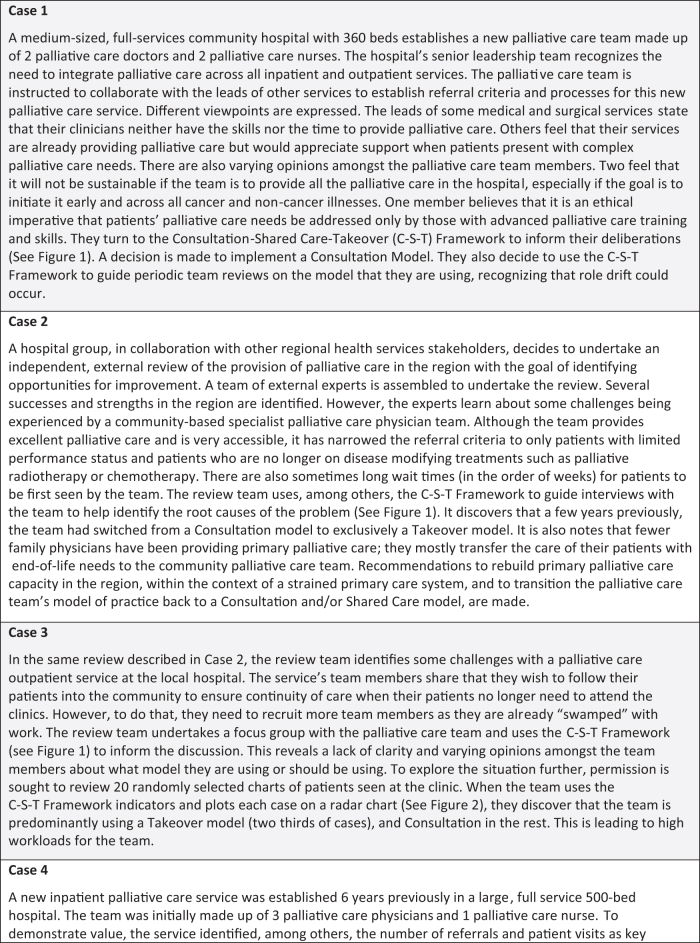

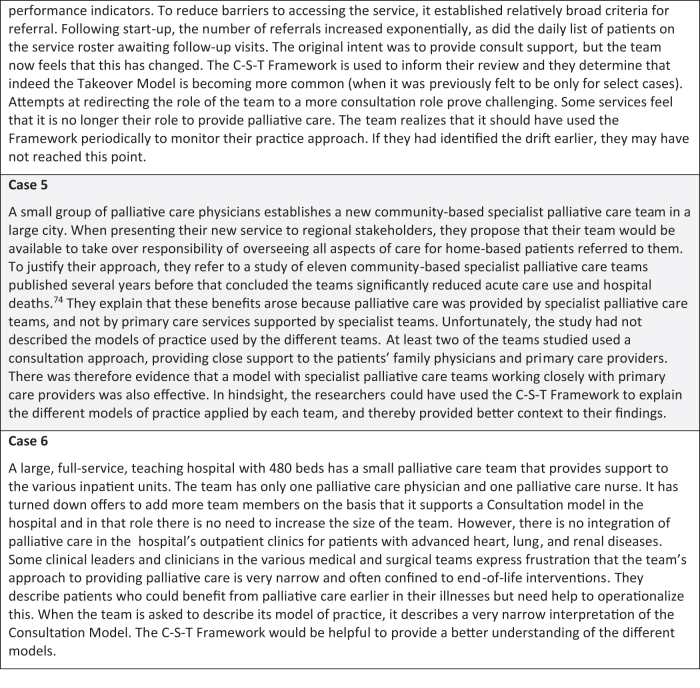
Illustrative cases to explain the role and application of the C-S-T Framework. (The cases are inspired by real life experiences; details modified for illustrative purposes).

Figure 3 provides several illustrative cases to describe the applications and uses of the C-S-T Framework.

## Discussion

### Main findings

The C-S-T Framework outlines that there are three major methods (or models) that specialist palliative care teams can interact with referring primary care or other specialist services. The Framework places these three, namely *Consultation*, *Shared Care*, and *Takeover* models, along a spectrum and provides descriptors across five domains, to help identify which model a clinician or team is using. Emerging evidence from the symposium and the studies that used it, especially the study by Maybee and colleagues,^36^ supports its face validity.

The practice model adopted by community- and hospital-based specialist palliative care clinicians and teams may have significant intended or unintended consequences for patients, the health care system, and themselves. The Framework serves as a tool for clinicians, managers, planners, and researchers to describe, discuss, understand, categorize, and monitor the models of practices adopted by these services. It provides a common language and understanding for the models.

### Contribution of the framework

The C-S-T Framework complements previous work in this area by providing more details on different models and situating them relative to each other.^2,24^ The Framework helps further characterize and study the role of specialist teams within service models such as liaison, integrated care, managed clinical networks, and pop-up services.^10^

Each model has its respective strengths, limitations, motivations, and impact. In the spirit of quality improvement, the Framework is meant to generate honest and open self-reflection. It is not meant to provoke competition between specialist and primary palliative care providers or assign blame for the adoption of one or another model. The goal is to use it to organize services using a systems approach, in a way that harnesses the expertise and roles of primary and specialist palliative care providers.^69,70^ The goal should be quality palliative care for individuals, aligned with their needs, and maximum access at a population level.^71–73^

Role drift may occur, necessitating periodic monitoring. A team may start with a *Consultation* mission but over time, for reasons within or outside its scope of influence, turn to *Takeover*. A *Consultation* team may find itself consistently applying an excessively narrow or inflexible interpretation of *Consultation*. Once a model is entrenched, changing to another model may require considerable effort and create tensions within the team and with other services. Although some flexibility is needed regarding which model to apply,^24^ confusion may arise with excessive or inconsistent flexibility.^34^ The Framework may help teams articulate their mission and what is expected of team members. It also helps them articulate their role to referring services.

Many factors may influence which model a clinician or service adopts. Internal drivers may include job satisfaction, efficiency, locus of control, team size, choice of remuneration model, and self-identified performance indicators. External drivers may include performance indicators, system expectations to demonstrate value added, and the conditions that support or impede the capability and the willingness of primary care practitioners to provide primary palliative care. In a Canadian study by Howard et al., for example, the remuneration method of the physicians influenced the model provided; a fee for service model where a physician is paid through clinical billings to government for clinical services rendered appeared to drive a *Takeover* model.^37^

The Framework has important research applications. It helps researchers describe models practiced by clinicians and teams when studying the impact of services. Seow and colleagues, for example, explored the impact of 11 community-based palliative care teams in the province of Ontario, Canada.^74^ Although they described the constitution of each team in terms of professions and number of members, they omitted to report that 2 of the 11 teams used *Consultation* models, while the others largely practiced *Takeover*; the 2 *Consultation* teams were among those that showed significant reductions in emergency department visits and hospitalizations.

Understanding the models is critical for workforce planning. Specialist workforce planning models may assume a *Consultation* model.^75^ If all specialist and primary-level palliative care is provided only by palliative care specialists, then much higher clinician numbers would be needed. When barriers exist to primary palliative care, strategies should be sought to address these rather than simply reverting to *Takeover*.^15,76^ Successful education programs have, for example, been described to address training gaps among primary care providers.^77^

### Strength and limitations

The Framework has evolved over time with input from many experts and sources and has been applied in real life. Some limitations of the Framework are noted. First, it has largely evolved in Canada. However, we feel that the concepts described in the Framework are applicable in many other jurisdictions and international experts and literature contributed to its early and more recent conceptualizing, Second, it has not been subjected to a large validation process involving large numbers of participants. Third, the Framework has not been applied large-scale across health care systems.

## Conclusion

The C-S-T Framework can be used to better describe, understand, assess, and monitor models of care being used by specialist palliative care clinicians and teams in their interactions with primary care providers and other specialist services. This is important because the modes of practice that specialist palliative care teams adopt has many implications for patients, the referrers, the health care system at large, and the palliative care clinicians themselves. Understanding and monitoring the roles of teams and clinicians using a tool such as the C-S-T Framework can support the development of strategies for the long-term sustainability of a service, its impact at community and individual patient levels. Further research is needed to explore the Framework and its models more broadly, the indicators that differentiate the models, and the application of the Framework at a larger health systems level, especially in different jurisdictions.

## Data Management and Sharing

There are no data available to share. The external review that is referred to in the Results section was undertaken confidentially and cannot be shared as the data would reveal identities of individuals and the system reviewed.

## Abbreviations Used

AMA: American Medical Association
C-S-T: consultation-shared care-takeover
MRP: most responsible practitioner
WHO: World Health Organization
